# High PEEP extubation as guided by esophageal manometry^☆^^☆☆^

**DOI:** 10.1016/j.rmcr.2024.101985

**Published:** 2024-01-23

**Authors:** Kathryn M. Pendleton, Jacob Fiocchi, Julia Meyer, Alexandra Fuher, Sarah Green, William M. LeTourneau, Ronald A. Reilkoff

**Affiliations:** a Division of Pulmonary, Allergy, Critical Care and Sleep Medicine, Department of Internal Medicine, University of Minnesota Medical School, Minneapolis, MN, USA; b Department of Internal Medicine, University of Minnesota Medical School, Minneapolis, MN, USA; c University of Minnesota Medical School, University of Minnesota, Minneapolis, MN, USA; d MHealth-Fairview Southdale Hospital, Edina, MN, USA; e Department of Anesthesiology and Perioperative Medicine, Respiratory Therapy, Mayo Clinic College of Medicine and Science, Rochester, MN, USA

**Keywords:** Ventilator weaning, Obese, Morbid obesity, Esophageal manometry, Positive end expiratory pressure (PEEP), Spontaneous breathing trials (SBT)

## Abstract

The ventilatory management of morbidly obese patients presents an ongoing challenge in the Intensive Care Unit (ICU) as multiple physiologic changes in the respiratory system complicate weaning efforts and make extubation more difficult, often leading to increased time on the ventilator. We report the case of a young adult male who presented to our ICU on two separate occasions with hypoxemic respiratory failure requiring intubation. Esophageal manometry (EM) guided positive end expiratory pressure (PEEP) titration was utilized during both ICU admissions to improve oxygenation and aid in extubation with spontaneous breathing trials performed on higher-than-normal PEEP settings and successful liberation on both occasions.

## 1 Introduction

Obese and morbidly obese patients (BMI >35 kg/m2) represent 7% of Intensive Care Unit (ICU) admissions and present unique diagnostic and management challenges [1]. Alterations in respiratory mechanics and chest wall physiology provide added difficulty in managing and weaning these patients from invasive mechanical ventilation. Clinically, this manifests as increased incidence of atelectasis and hypoxemia which may translate to difficulties weaning from mechanical ventilation, extended duration of ventilatory support, and need for tracheostomy [2,3]

Positive end-expiratory pressure (PEEP) is commonly used in mechanical ventilation to improve oxygenation and reduce alveolar derecruitment. Morbidly obese patients may be especially reliant on PEEP due to their decreased functional residual capacity (FRC), and studies have shown that application of PEEP can improve respiratory mechanics in this population [4]. Standard mechanical ventilator maneuvers for monitoring airway pressures (Paw) can be unreliable in morbidly obese patients because they depend on measured Paw to estimate alveolar distension, and Paw alone cannot distinguish between poor lung compliance and poor chest-wall compliance [5]. Esophageal manometry (EM) can be used to measure esophageal pressure (Pes) and better estimate transpulmonary pressure (P_L_ = P_aw_-P_es_), allowing for optimal PEEP titration. EM-guided PEEP titration typically results in the application of higher PEEP, which has been shown to improve oxygenation and respiratory system compliance during mechanical ventilation in morbidly obese patients [4]. The use of higher PEEP for ventilatory weaning and spontaneous breathing trials (SBT) in morbidly obese patients is unknown.

We herein report a case of a patient who twice presented with severe acute hypoxemic respiratory failure requiring mechanical ventilator support where EM-titrated PEEP was employed utilizing higher-than-average PEEP settings that were continued during facilitated successful liberation trials.

## 2 Case presentation

**Fig. 1:**
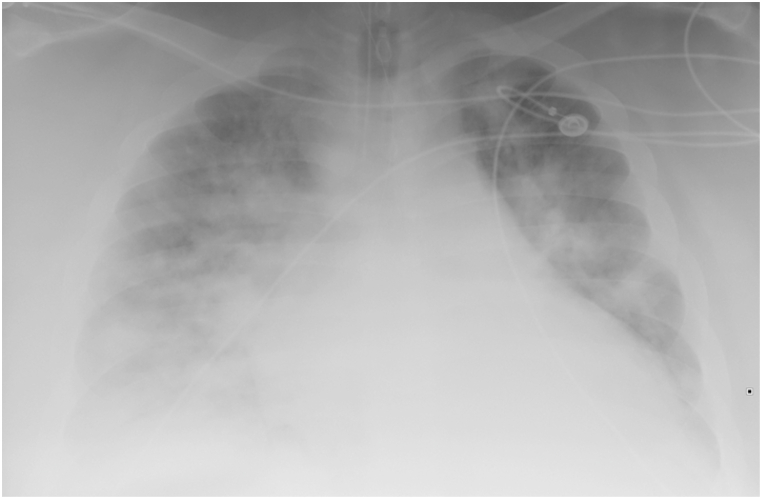
Pre EM Titration. Marked cardiac enlargement with increased pulmonary vascularity. Prominent diffuse bilateral pulmonary infiltrates. Remainder unremarkable.

**Fig. 2:**
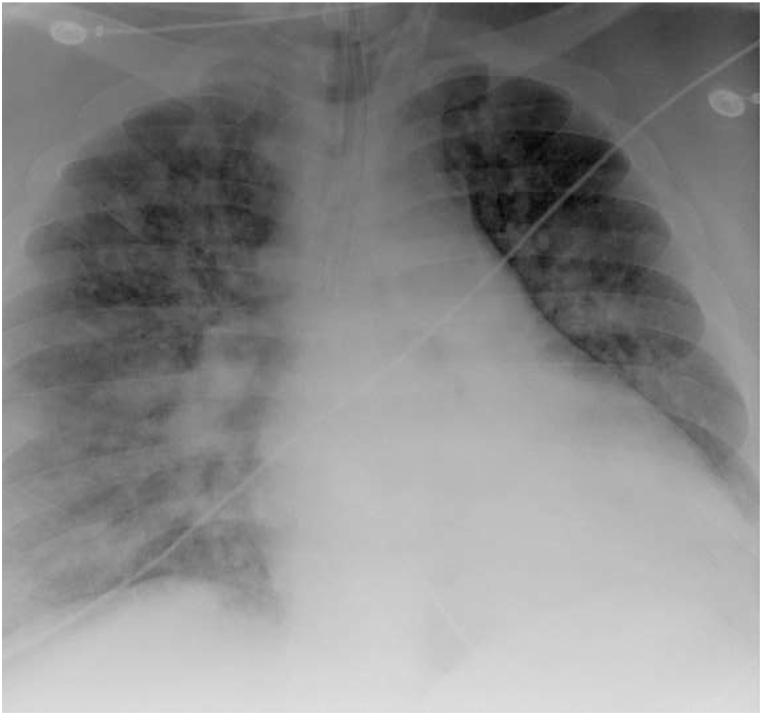
Post EM Titration. Endotracheal tube tip is about 3.9 cm above the carina. NG tube overlies the mediastinum, the tip not seen, and can be confirmed by abdominal radiograph. Diffuse bilateral patchy airspace disease, similar to prior. No large effusions or pneumothorax. Unchanged cardiac size.

A 22-year-old man with a history of morbid obesity (BMI 79.4), obstructive sleep apnea (intolerant of CPAP), and hypertension presented to the emergency department with a week-long prodrome of fever, productive cough, chest pain, and progressive shortness of breath. Initial vital signs showed a respiratory rate 46, oxygen saturation 78% on room air, temperature 37.9 °C, blood pressure 77/57 mm Hg, and heart rate 156. Initial examination was notable for tripod positioning, distant breath sounds bilaterally and significantly labored breathing ultimately requiring intubation. Post intubation arterial blood gas confirmed hypercapnic and severe hypoxemic respiratory failure with pH 7.18, pCO_2_ 72, and paO_2_ 81 on 100% Fi0_2_ (Pa0_2_/FiO_2_ of 81). Chest x-ray obtained after intubation revealed vascular congestion with cardiomegaly versus hypoaeration of the lungs with an enlarged heart and retrocardiac opacity, left lower lobe atelectasis or possibly pneumonia (Figure 1 Pre EM Titration). Initial laboratory studies showed white blood cell count of 27, hemoglobin 15.6, lactic acid 3.6, BNP 656, procalcitonin 6.1, and negative troponin. He was started on Piperacillin-Tazobactam and Vancomycin and bolused 500 ml crystalloid with improvement in his hemodynamics. Upon arrival to the ICU his initial ventilatory settings of Assist Control Ventilation (AC): rate of 25, tidal volume (V_T_) 460 ml, PEEP of 12 cm H_2_O and 100% FIO_2_. EM was initiated per ICU protocol; the transpulmonary (P_L_) PEEP was −16 cm H2O and the transpulmonary (P_L_) plateau was −7 cm H2O. Ventilator settings were adjusted with increases in both Vt and PEEP to 550 ml and 26 cm H2O, respectively, with P_L_ PEEP improving to −2 cm H2O and P_L_ plateau to 5 cm H2O. Serial EM assessments and ventilatory support were down-titrated through his ICU course as his respiratory failure continued to improve on empiric antibiotics and steroids (Figure 2 Post EM Titration). Infectious disease workup was positive for streptococcus pyogenes bacteremia with suspicion for streptococcus pharyngitis. His respiratory viral panel returned positive for adenovirus. Pressure support (PS) with inspiratory pressure augmentation of 20 cm H20 over PEEP 15 (PS 20/15) was initiated on hospital day 7 with the patient successfully extubated to bilevel positive airway pressure ventilation on hospital day 10 after passing a spontaneous breathing trial on PS 15 cm H2O, PEEP 10 cm H2O (comparable BIPAP settings.) He was discharged to inpatient rehabilitation on hospital day 16.

**Fig. 3:**
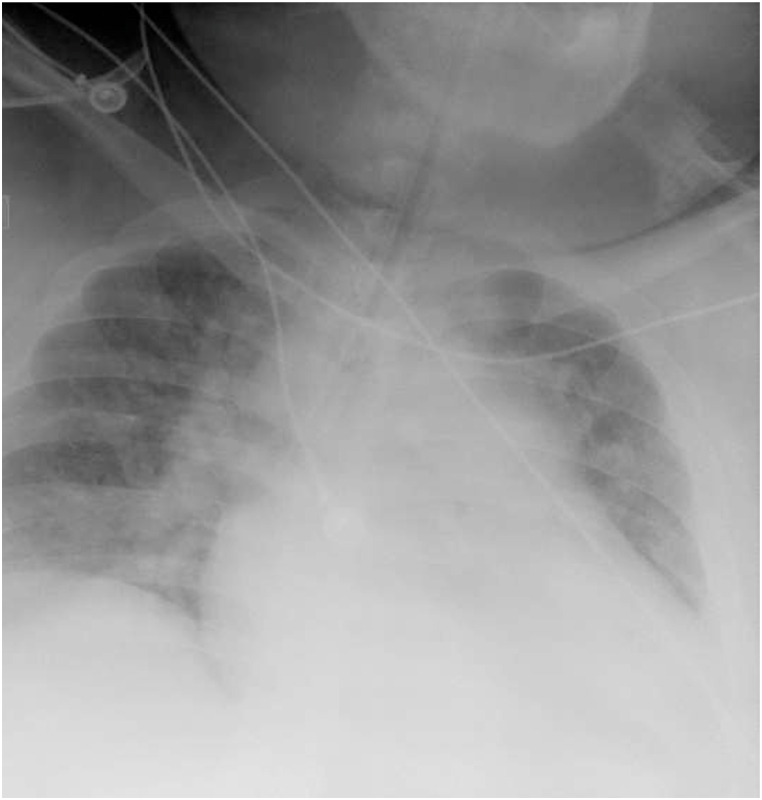
Pre EM Titration (admit 2). Portable view of the chest is performed. Endotracheal tube is in the right mainstem bronchus and should be repositioned approximately 2–3 cm in the more proximal airway. Pulmonary vascular congestion is present possibly due to hypoaeration of the lungs. Heart appears enlarged. Retrocardiac opacity likely due to left lower lobe atelectasis or possibly pneumonia.

**Fig. 4:**
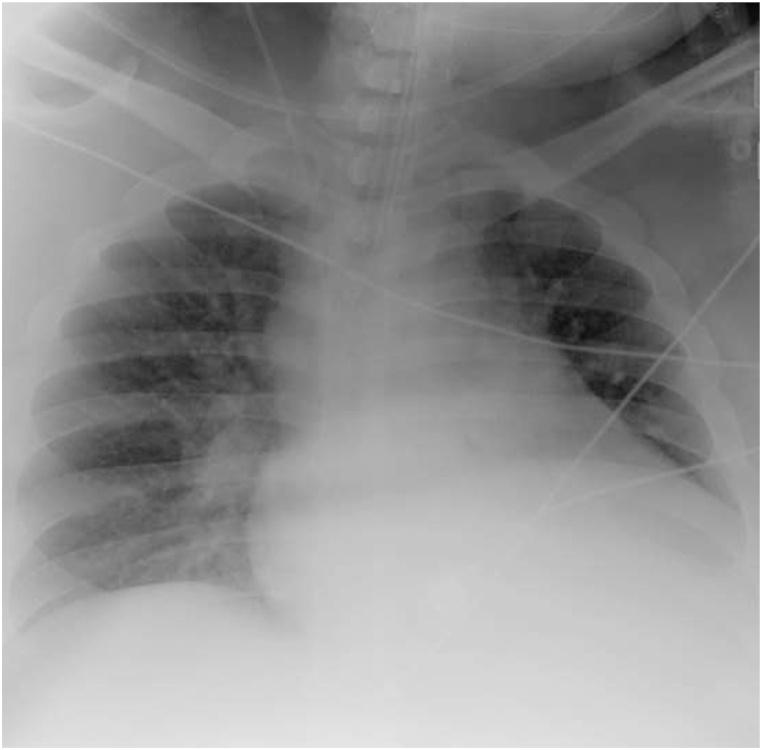
Post EM Titration (admit 2).Marked left lower lobe atelectasis and/or infiltrate. Right lung grossly clear. Tubes and lines grossly unchanged.

This same patient, presented to the same hospital a few years later with a week-long history of increased dyspnea, chest tightness, fever, and cough. His history was notable for Covid −19 exposure, though the patient recounted a negative test earlier in the week. His initial saturations on room air were 57%, respiratory rate 36, temperature 37.1 °C, blood pressure 117/52 mm Hg, and heart rate 104. His exam was notable for conversational dyspnea, tachypnea, and decreased breath sounds throughout. Chest x-ray revealed marked cardiac enlargement (Figure 3. Pre EM Titration (admit 2) with diffuse bilateral infiltrates. EKG was notable for right ventricular hypertrophy, which was new from his prior admission, troponin was negative. Venous blood gas was pH 7.38, pCO_2_ 49. He was briefly trialed on non-invasive ventilation with 100% FIO2 but continued to decline, ultimately requiring intubation and mechanical ventilation for increased work of breathing and hypoxemia. Post-intubation, the patient experienced significant decruitment with desaturation into the 40's. He had modest recovery on pressure control though still requiring bag-mask ventilation and high-level ventilating pressures (Phigh 37 cm H2O, PEEP 20 cm H_2_O), deep sedation, and paralysis with inhaled epoprostenol to recover his oxygen saturations into the high 80's -low 90's. He was empirically started on dexamethasone given high clinical suspicion for Covid −19 infection. Upon arrival to the ICU, he was transitioned back to volume control mode with settings AC 20, Vt 500 ml, PEEP 20 cm H_2_O and FiO_2_ 100% PEEP titration and recruitment via esophageal manometry (EM) was again initiated per ICU protocol. His initial transpulmonary (P_L_) PEEP was −14 cm H2O and transpulmonary (P_L_) plateau was 11 cm H_2_0. Ventilator settings were adjusted, with PEEP titrated to 28 cm H_2_O and V_T_ 550 ml resulting in P_L_ PEEP improvement to −4 cm H_2_O without change in P_L_ plateau. Repeat imaging demonstrated improved aeration (Figure 4 Post EM Titration (admit 2), which correlated with the patient's decreased oxygenation requirements, facilitating rapid discontinuation of inhaled epoprostenol and improvement in his PaO_2_/FiO_2_ ratio which precluded the need for prone positioning. Admission Covid −19 testing returned positive, and the patient was treated with a full course of Dexamethasone, Remdesivir, convalescent plasma and a single dose of Tocilizumab. Serial EM maneuvers were performed, and ventilatory support continued to be titrated correlating with the patient's clinical improvement. Spontaneous breathing trials with higher levels of PEEP (14–16 cm H_2_O) were initiated on hospital day 10. On hospital day 12, the patient self-extubated on a setting of PS with inspiratory pressure augmentation of 14 cm H2O and PEEP 10 cm H2O, with saturations 89% on room air. He was placed on empiric bilevel noninvasive ventilation at 24/10 and 6 L FIO_2_ for continued support though he reported no respiratory distress. He was eventually transferred out of the ICU on hospital day 15 and discharged on hospital day 19. A summary of the patients mechanics pre and post esophageal manometry from both admissions is supplied in Table 1.

**Table 1:** Airway pressures pre and post esophageal manometry.

		Vent Setting (AC:VC)	Extrinsic PEEP cm H20	Plateau Pressure cm H20	Transpulmonary PEEP (P_L_ PEEP) cm H20	Transpulmonary Plateau (P_L_ Plateau) (cm H20)	True Driving Pressure	P/F Ratio (on 100%)
1st ADMISSION	Initial Settings	TV460 ml	12	33	−16	−7	9	81
		FIO2% 100%						
	EM Adjustment	TV 550 ml	26	39	−2	5	7	141
		PEEP 26						
		FIO2% 100%						
2nd ADMISSION	Initial Settings	TV500 ml	20	Not measured (MAP 37, Peak 57)	−14	11	25	53^a^ (S/F ratio 87)
		PEEP 20						
		FIO2% 100%						
	EM Adjustment	TV550 ml	28	33 (MAP 32, Peak 41)	−4	11	15	74
		PEEP 28						
		FIO2% 100%						

Abbreviations: EM – Esophageal Manometry; AC:VC – Assist Control-Volume Control; PEEP – Positive End Expiratory Pressure; TV – Tidal Volume; MAP – Mean Airway Pressure.

a ABG not measured in emergency department, imputed from O2 Saturations and https://opencriticalcare.org/imputed-pao2-calculator/.

## 3 Discussion

There is a continuing debate regarding the accuracy and clinical efficacy of using EM for optimal PEEP titration, with even fewer studies evaluating the role of EM in assisting medical decision-making with respect to weaning and liberation from mechanical ventilation. Current ventilator liberation guidelines do not specifically designate an acceptable range of PEEP for SBTS, although many safety screens suggest that PEEP should be ≤ 8 H_2_O before conducting an SBT [6]. One study in morbidly obese patients suggests that the most accurate assessment of post-extubation work of breathing is either a t-piece trial or SBT with pressure augmentation of 0 and PEEP 0 [7]. This contrasts with case reports and other studies, which have shown that even a transient withdrawal of PEEP during weaning, can precipitate large inspiratory pressure swings, increasing work of breathing and the development of atelectasis in morbidly obese patients, potentially leading to weaning failure [8,9]. In tracheotomized, morbidly obese patients, using EM-guided PEEP titration to overcome negative transpulmonary pressure was safe and resulted in more rapid weaning from mechanical ventilation among patients who successfully weaned, suggesting a benefit to use [10].

Esophageal manometry can be a useful tool in the ICU for estimating pleural pressures and calculating transpulmonary pressures, however there are important limitations with its use. Esophageal pressure measurement (P_es_) most closely reflects pleural pressures in the dependent to middle lung regions [11]. Therefore measurements may not accurately reflect pleural pressures across all lung regions, with notable differences between dependent and non-dependent zones (i.e., lung bases may more negative pleural pressure as compared to lung apices which may have less negative pleural pressures). These regional differences may be exacerbated by clinical conditions such as atelectasis or positional changes (supine vs prone, etc.). These factors need to be taken into consideration when using EM-derived data and integrating it with other clinical data in order to make well-informed mechanical ventilation and patient management decisions.

Our case highlights a real-world application of EM, as we believe that extubating morbidly obese patients on higher than conventional levels of PEEP occur in clinical practice and that using physiologically guided, higher than conventional levels of PEEP during SBTs is safe and feasible for successful extubation in these patients. We eagerly anticipate larger studies in this area, such as the upcoming “Providing Optimal PEEP during Mechanical Ventilation for Obese Patients Using Esophageal Balloon” (PROP-OPEN; NCT03951064) trial. This study, enrolling mechanically ventilated patients with BMI >40, should better elucidate the benefits and limitations of EM-guided PEEP titration and weaning of ventilatory support in patients with morbid obesity.

## 4 Conclusion

Our case demonstrates the clinical utility of and application of a higher level of PEEP support during mechanical ventilation of morbidly obese patients. We also suggest that some morbidly obese patients may benefit from SBTs done on higher than conventional levels of PEEP and this practice may better facilitate successful liberation from mechanical ventilation.

## Funding

No financial support was provided for the study.

## CRediT authorship contribution statement

**Kathryn M. Pendleton:** Conceptualization, Formal analysis, Investigation, Writing – original draft, Writing – review & editing. **Jacob Fiocchi:** Writing – original draft, Writing – review & editing. **Julia Meyer:** Investigation, Writing – review & editing, Data curation. **Alexandra Fuher:** Data curation, Investigation. **Sarah Green:** Data curation, Investigation, Writing – review & editing. **William M. LeTourneau:** Writing – review & editing. **Ronald A. Reilkoff:** Conceptualization, Data curation, Formal analysis, Investigation, Methodology, Supervision, Writing – original draft, Writing – review & editing.

## Declaration of competing interest

The authors declare that they have no known competing financial interests or personal relationships that could have appeared to influence the work reported in this paper.
